# Symptoms of autism in Williams syndrome: a transdiagnostic approach

**DOI:** 10.1038/s41598-024-68089-0

**Published:** 2024-07-30

**Authors:** Charlotte Willfors, Jacqueline Borg, Johan Lundin Kleberg, Astrid Hallman, Marcus Van Der Poll, Karl Lundin Remnélius, Hanna Björlin Avdic, Sven Bölte, Ann Nordgren

**Affiliations:** 1 Department of Molecular Medicine and Surgery, Karolinska Institute, Stockholm, Sweden; 2 Department of Clinical Genetics and Genomics, Karolinska University Laboratory, Karolinska University Hospital, Stockholm, Sweden; 3 Department of Clinical Neuroscience, Centre for Psychiatry Research, Karolinska Institute, Stockholm Health Care Services, Stockholm, Sweden; 4 Department of Clinical Neuroscience, Centre for Cognitive and Computational Neuropsychiatry, Karolinska Institute, Stockholm Health Care Services, Stockholm, Sweden; 5 Neuropsychiatry, Sahlgrenska University Hospital, Gothenburg, Sweden; 6 Department of Psychology, Stockholm University, Stockholm, Sweden; 7 Center of Neurodevelopmental Disorders (KIND), Centre for Psychiatry Research, Department of Women’s and Children’s Health, Karolinska Institute, Stockholm Health Care Services, Stockholm, Sweden; 8 Child and Adolescent Psychiatry, Stockholm Health Care Services, Stockholm, Sweden; 9 Curtin Autism Research Group, Curtin School of Allied Health, Curtin University, Perth, Australia; 10 Department of Clinical Genetics and Genomics, Sahlgrenska University Hospital, Gothenburg, Sweden; 11 Department of Laboratory Medicine, Institute of Biomedicine, University of Gothenburg, Gothenburg, Sweden

**Keywords:** Genetics, Psychology, Diseases, Health care, Signs and symptoms

## Abstract

Williams syndrome (WS) is associated with atypical social communication and cognition reminiscent of the behaviours observed in autism. Nonetheless, WS also differs significantly from autism, such as regarding social motivation, which is typically enhanced in WS and reduced in autism. This study sought to examine the conditions’ transdiagnostic similarities and differences for autistic symptoms and social functioning, and their developmental trajectories, by comparing individuals with WS (n = 24) and those diagnosed with idiopathic autism (n = 24) and attention deficit hyperactivity disorder (ADHD; n = 24), aged 9 to 53 years, on measures of autism, social functioning, IQ and cooccurring psychiatric conditions. Although only 12.5% in the WS group met the criteria for an autism diagnosis, a majority exhibited distinct difficulties within social communication, social cognition, repetitive behaviours, and atypical sensory reactivity resembling autism. Conversely, elevated social motivation and a high number of social initiatives accompany these characteristics. No group differences in the developmental trajectories of autism symptoms were found. Our results demonstrate that autistic behaviours are more frequent in individuals with WS, than in individuals with idiopathic ADHD, and emphasize the need for clinical management of these behaviours.

## Introduction

Williams syndrome (WS) is a rare genetic neurodevelopmental condition caused by a deletion of 26–28 genes on chromosome 7q11.23^1^, with an estimated prevalence of 1 in 7500 live births^2^. Distinct medical, cognitive and behavioural features characterize the phenotype of WS. The neuropsychological profile is defined by a mild to moderate intellectual disability, with relative strengths in verbal ability and difficulties with visuospatial and executive functions^3^.

Individuals with WS are typically described as hypersocial, loquacious, empathic, and friendly. However, the behavioural phenotype in WS is complex. The generally outgoing and socially fearless personality is often combined with impairments in reciprocal social interaction, hyperacusis and non-social anxiety^4^, symptoms that are reminiscent of autism [or autism spectrum disorder, the formal term used in the American Psychiatric Association's Diagnostic and Statistical Manual of Mental Disorders, 5th edition (DSM-5) and WHO's International Classification of Diseases, 11th revision (ICD-11)]. Children as well as adults with WS often experience social challenges leading to social isolation^5,6^, and in comparison with other syndromes including intellectual disability, such as Down syndrome, individuals with WS have more difficulties establishing and maintaining social relationships^7^. Both social communication challenges, restricted and repetitive behaviours (RRB) and altered sensory reactivity have been found in WS^8^. There seems to be an improvement in social difficulties with age in WS, which is not the case for example for Prader-Willi syndrome^9,10^, although these findings are not conclusive^11^. Unique characteristics of the WS social phenotype, not overlapping with autism, have also been described. These include an elevated attraction to strangers^12^, a preference to direct attention to other people’s faces^13^, and insensitivity to negative emotional signals^14^. Interestingly, although individuals with WS demonstrate increased attention to others’ faces, they exhibit reduced orienting to the eye region in comparison to typically developed individuals and respond to eye contact with lower physiological arousal than typically developed individuals^15,16^. To conclude, the autistic features associated with WS might be captured by the subgroup of autistic individuals originally described by Wing and Gould as “active but odd”^17^. Autistic individuals categorised as “active-but-odd” actively seek interaction with others, although in unusual ways, such as by standing too close to a conversation partner or holding a monolog about a particular interest.

Autistic traits and social challenges are also common in individuals with attention deficit/hyperactivity disorder (ADHD), and two-thirds of children with WS meet the criteria for an ADHD diagnosis^18^. This overlap of symptoms between the different diagnostic categories, favours a transdiagnostic perspective when studying autism^19^. Therefore in the current study, a transdiagnostic approach was applied, meaning that we investigate commonalities as well as specific profiles of autistic characteristics across diagnostic categories.

The Autism Diagnostic Observation Schedule-2 (ADOS-2) and previous versions (ADOS-G), in combination with the Autism Diagnostic Interview-Revised (ADI-R), are widely accepted international standards for the assessment of autism^20–22^. Nevertheless, only a few studies in the WS literature have used the ADOS or ADI-R^23–25^, and only one study combined the two instruments^26^. These studies suggest that 10 to 50% of children with WS meet the cut-off criteria for autism or autism spectrum disorders on these scales. One of the studies was based on a cross-syndrome design and included matched control groups of children with autism, pervasive developmental disorder (not otherwise specified), and non-autistic developmental conditions of mixed aetiology. The results showed that the WS group displayed more social interaction difficulties than did the non-autistic group, indicating greater socio-communicative impairment than expected based on developmental delay alone^24^. The largest study included 100 children with WS (aged 3 to 15 years) and found that 30 to 35% of the sample met the cut-off for autism spectrum according to the ADOS^27^. Symptoms separating individuals with WS classified as autistic or non-autistic were overall quality of social overtures and response, eye contact, conversation skills, and speech alterations. However, there are also difficulties that are common in WS, such as reduced empathy insight, and reduced imagination and creativity, which are not related to autism classification^27^. To date, most studies on autistic symptomatology in individuals with WS have been based on children, and to the authors’ best knowledge, no study has used the ADOS in adolescents and adults with WS and fluent speech.

In one small study (n = 9), the ADOS and ADI-R were combined for the assessment of autism in individuals with WS^26^. Although the sample ranged in age from 4 to 39 years, only module 1 was used, mostly applied to children, due to low language abilities. The participants were all first diagnosed with autism and subsequently found to have WS, thus making it a selective group with more severe autism symptoms than the general WS population. The sample presented severe impairments in social interaction and communication (including the absence of expressive language), as well as severe RRB symptoms. The results suggested an improvement in social communication ability with increasing age, while the results regarding RRB symptoms were inconclusive^26^.

The Social Responsiveness Scale 2nd edition (SRS-2) is a quantitative measure of autistic traits^28^, and is a frequently used measure of social awareness, motivation, cognition and communication in individuals with WS (e.g.^5,29–31^). These studies showed that the majority of children with WS are classified as having mild to moderate difficulties in social functioning. The greatest challenges are observed in areas related to social cognition and flexibility, while high scores in prosocial domains such as social motivation and social awareness are less common. According to a transdiagnostic comparison between WS and autism using the SRS, the WS group lacked awareness of personal space boundaries, which was not found in autism or typically developing control groups^30^. Riby and colleagues investigated the association between social functioning as measured by SRS, and anxiety in a sample of mixed-aged individuals with WS (6 to 36 years). These authors showed a positive association between anxiety and social impairments. By splitting the WS group into high- and low-anxiety groups, they found that the high-anxiety group had significantly more impairments than the low-anxiety group related to social awareness, social cognition and social communication but did not differ in social motivation or flexibility^29^.

Some characteristics of ADHD are shared with autism, and the two disorders have similar underlying neuropsychological variations (i.e. executive functions, processing speed, language delay, perception, and emotion regulation)^32,33^. Nevertheless, autism and ADHD differ considerably regarding symptom profiles^34^. Hence, individuals with ADHD constitute an ideal comparison group for distinguishing between general social behaviour alterations associated with neurodevelopmental disorders, and specific autism symptomatology. To disentangle shared and syndrome-specific characteristics across common neurodevelopmental conditions and WS, we compared individuals with WS to those diagnosed with idiopathic autism (i-autism) and idiopathic ADHD (i-ADHD) in a transdiagnostic design. Standardized measures of autism, including the ADOS-2, ADI-R and SRS-2, were collected to assess autistic symptoms and social behaviour in children and adults.

The primary aim was to identify syndrome-specific profiles of autistic symptoms associated with WS. To perform a more detailed investigation of social cognition and social approach behaviour, specific items were also analysed. Since previous research has suggested that WS is associated with atypical attention toward others’ eyes^13,15^, excessive desire to approach others, indiscriminate trust toward others, and difficulties understanding subtle social cues^30,31,35^, items possibly capturing these areas were examined specifically. Second, we investigated whether symptom severity changed over time (i.e., from age 4 to 5 years or ever to current age) in WS patients as well as in control groups, since there are mixed results regarding this topic in the literature^10,11,26^.

## Methods and materials

### Participants

Twenty-four individuals with WS were recruited from all over Sweden via interest organizations and health care facilities. The WS diagnoses were confirmed through genetic testing within the study or confirmation of previous genetic testing via medical records. The participants were accompanied by at least one parent and attended a two-day examination at the Karolinska University Hospital, Stockholm, Sweden, including clinical interviews, neuropsychological tests, biological sampling, and photography. All WS participants were verbally fluent.

The comparison samples were collected from the Roots of Autism and ADHD Twin Study in Sweden (RATSS)^36,37^. Data from more than 400 twins were collected via the RATSS, and the twins participated in comprehensive assessments, including the ADOS-2, ADI-R, SRS-2, and Wechsler scales and clinical interviews for the assessment of psychiatric comorbidity. Equal sample sizes were selected for the control samples to avoid interfering with the robustness of the equal variance assumption in the calculations of one-way ANOVAs. Hence, 24 single twins with i-autism (and no co-occurring ADHD) and 24 single twins with i-ADHD (and no co-occurring autism), were selected from the total RATSS sample. The twins closest in age and sex to each WS participant were selected.

#### Ethical declarations

All the subjects and their parents provided informed consent for inclusion before they participated in the study. The study was conducted in accordance with the Declaration of Helsinki, and the protocol was approved by the national Swedish or responsible regional ethical review board (2018/1218-31 and 2016/1452-31).

### Measures

#### Neurodevelopmental and psychiatric diagnoses

A consensus diagnosis of autism was supported by the ADOS-2 and ADI-R. The diagnosis of ADHD was determined using information from multiple sources. For participants < 18 years, parents were interviewed with the Mini International Neuropsychiatric Interview for Children and Adolescents (MINI KID; the WS sample)^38^ or the Kiddie Schedule for Affective Disorders and Schizophrenia (K-SADS; the i-ADHD and i-autism samples)^39^. For participants 18 years or older, parents and participants were interviewed using the MINI (the WS sample)^38^ or the Structured Clinical Interview for DSM-IV-Axis I Disorders (SCID-I, the i-ADHD and i-autism samples)^40^ combined with the Diagnostic Interview for ADHD in adults (DIVA)^41^. A psychologist or psychiatrist, with adequate training in the specific instruments, administered the clinical assessments.

#### General intellectual ability

General intellectual ability was assessed with the Wechsler Intelligence Scales, with the exception of one participant with limited verbal ability, whose intellectual ability was assessed with the Leiter International Performance Scale, 2nd edition. Since data collection in the RATSS started before the fifth version of the Wechsler Intelligence Scale for Children (WISC-V) was available in Sweden (i.e. 2011), the WISC-IV was used for the assessment of participants with i-autism and i-ADHD up to 16 years of age (n = 22)^42^. The younger participants in the WS sample, for whom the data collection took place later in time, were assessed with the WISC-V (n = 4)^43^. All participants older than 16 years were assessed with the Swedish version of the fourth version of the Wechsler Adult Intelligence Scale (WAIS-IV) (n = 45)^44^. The General Ability Index (GAI) was used to estimate full-scale IQ from the Wechsler scales, and the IQ screening was used for the Leiter scale. The visual spatial index from the WISC-V are reported as equivalent to the performance index in the other versions (i.e., the WISC-IV and WAIS-IV) since they have been shown to have the highest correlation with the performance index in comparisons between different Wechsler versions (r = 0.78)^45^.

#### Autistic symptoms and social functions

The ADI-R is an extensive semi-structured parental interview that captures general background, family, medical, and educational history, and a detailed account of autistic symptoms in three domains: communication, reciprocal social interaction, and RRB. The algorithm of the ADI-R is based on groups of items within each of the three key areas to correspond with diagnostic criteria for autism in the ICD-10 and DSM-IV. The results of the interview may be used in two ways. The first and most common use of the ADI-R is to apply a diagnostic algorithm for scoring, taking into account developmental issues, and it is based on the 4- to 5-year period of life or “ever”, with validated diagnostic cut-offs for each domain. This also includes items covering early developmental delays and/or signs of autism, e.g., delays in language development and the time point of the first signs of autism. Second, it can be used as a current algorithm for assessing the current behaviour of a person during the past months^46^. There are no recommended cut-offs for diagnoses for the current algorithm, and a general decrease in symptom severity between childhood and adolescence/adulthood has been reported in individuals diagnosed with autism when comparing the diagnostic and current algorithms^47,48^. The results from the diagnostic algorithm are here referred to as symptoms “during development”, and the results from the current algorithm are referred to as symptoms in “adolescence/adulthood”.

The ADOS-2 is a structured observation of individuals using a standardized semi-structured observation schedule involving interview questions and social activities. The instrument captures the quality and severity of social communication symptoms and the presence of RRB. It is sensitive to manifestations in individuals with a range of intellectual abilities. Ratings are made for specified aspects of language and communication, reciprocal social interaction, play/imagination, RRB and other atypical behaviours. The ADOS-2 is divided into five modules (toddler and module 1–4), and modules 3 and 4 are designed for children and adults who are verbally fluent^49^. In the present study, modules 3 (n = 10) and 4 (n = 61) were used, and the revised diagnostic algorithms were used for both modules^50^. The ADOS-2 score was missing for one participant with i-autism. For this participant, the autism diagnosis was supported by medical records and the ADI-R.

The parent-reported SRS-2 is a 65-item questionnaire that measures symptoms associated with autism^28^. The responses that caregivers provide about their children’s behaviours yield T scores on various scales of the SRS-2 as well as a total score. However, reliability studies suggest that the scale is mainly one- or two-dimensional ^51^, therefore, interpretation of the subscales should be performed with caution. In the present study, data from both the child version (aged < 18 years in the WS sample and aged < 19 years in the i-autism and i-ADHD samples) and the adult version were collected. The raw values were entered into the analyses as recommended for research purposes^28^, but T scores are reported for descriptive purposes. T scores below 60 indicate no clinically significant concerns, T scores of 60–75 indicate difficulties in the mild to moderate range, and T scores greater than 76 indicate severe levels of difficulties^28^.

#### Analysis of specific items

The following items measuring current behaviour in adolescence and adulthood were selected from the ADOS-2, ADI-R and SRS-2 for analysis: unusual eye contact (observed and parent report), Quality of social contact (observed and parent report), Amount of social initiatives (observed), Inhibited social behaviour (parent report), Inappropriate questions and behaviour (parent report), Awareness of others’ personal space (parent report), Awareness of someone taking advantage of him/her (parent report), and The ability to understand others’ tone, voice and facial expressions (parent report).

### Data analysis

Descriptive analyses were used to characterize and describe the samples. For normally distributed data, between-group comparisons were performed using one-way ANOVA (or Welch ANOVA if the assumption of homogeneity was not met) with Tukey post hoc tests. If the data were not normally distributed, the Kruskal‒Wallis test with a nonparametric post hoc test was used. Changes over time were investigated using percentage changes to be comparable with previous studies^47^.

We used Spearman’s correlation coefficient to explore associations between IQ and autistic traits and characteristics. For all comparisons, effect sizes are reported, and an alpha value of 0.05 was applied.

Since the groups differed in IQ (i.e., the WS group had a significantly lower IQ score than the i-autism and i-ADHD groups; see results below), including IQ as a covariate interfered substantially with the independent variable and decreased the group effect (for a detailed discussion, see^52^). Hence, IQ was not added as a covariate in the group comparisons; instead, the mean IQs for the different scoring options were included in supplementary material for a descriptive illustration of the effects of IQ on the outcome measures.

All analyses were conducted using SPSS version 28^53^. Study data were collected and managed using REDCap electronic data capture tools hosted at the Karolinska Institutet^54,55^.

## Results

### Descriptive statistics

Intellectual level, neurodevelopmental and psychiatric conditions were assessed within the three groups (Table 1). The associations between IQ and different outcome measures of autistic traits and symptoms were analysed; see the supplementary material for details (Table S1). There were no correlations between IQ and ADI-R scores during development in any of the diagnostic groups. In the i-autism group, there was a negative correlation between the current ADI-R score and total IQ, indicating that a higher IQ was associated with fewer social communication symptoms. This association was not found in the other diagnostic groups. There was a negative association between age and SRS scores in all three diagnostic groups. For the ADI-R and ADOS-2 scores, there was only a correlation between age and the ADI-R C RRB scores, with less RRB behaviour occurring in older individuals. There was no effect of sex/gender on any of the outcome measures. For 93% of the total sample, both biological parents were of Scandinavian origin.

**Table 1:** Demographic data and neurodevelopmental/psychiatric diagnoses.

	WS (N = 24)	I-autism (N = 24)	I-ADHD (N = 24)
Sex/gender % female	50%	50%	46%

	Mean (SD), range	Mean (SD), range	Mean (SD), range
Age	29.4 (13.4),9–53	19.5 (4.5),14–31	17.1 (4.9),13–36
Total IQ	56.71 (10.29), 44–81	92.13 (22.51), 48–142	97.33 (14.76), 64–129
Verbal IQ	65.29 (12.03), 45–97	94.59 (20.56), 64–134	92.54 (20.11), 34–130
Nonverbal IQ^a^	59.75 (8.42), 49–76	92.75 (20.38), 38–136	93.17 (25.23), 24–124

Diagnoses	N (%)	N (%)	N (%)
Intellectual disability—moderate	7 (29.2%)	1 (4.2%)	0
Intellectual disability—mild	15 (62.5%)	3 (16.7%)	1 (4.2%)
Autism	3 (12.5%)	24 (100%)	0
ADHD	4 (16.7%)	0	24 (100%)
Learning disorders	0	3 (12.5%)	6 (25%)
Communication disorders	0	0	2 (8.4%)
ODD	0	1 (4.2%)	3 (12.5%)
Anxiety disorders	15 (62.5%)	6 (25%)	8 (33.3%)
Depressive disorders	3 (12.5%)	3 (12.5%)	1 (4.2%)
OCD	0	3 (12.5%)	1 (4.2%)
Body dysmorphic disorder	0	1 (4.2%)	0
Tic disorder	1 (4.2%)	1 (4.2%)	0
Alcohol/substance use disorders	0	0	1 (4.2%)
Psychotic disorders	2 (8.4%)	0	0
Eating disorder	0	0	1 (4.2%)

*M* mean, *SD* standard deviation, *WS* Williams syndrome, r, *ADHD * attention deficit/hyperactivity disorder, *ODD* oppositional defiant disorder, *OCD* obsessive compulsive disorder.

^a^Performance index score on the WISC-IV and WAIS-IV and the visuospatial index score on the WISC-V are reported.

#### ADI-R

Since several scores were not normally distributed, medians and median absolute distances (MADs) were calculated, and the Kruskal‒Wallis H test was used to determine whether there were group differences in the ADI-R and ADOS-2 scores (Table 2). Pairwise comparisons were performed using Dunn's procedure with a Bonferroni correction for multiple comparisons^56^, and adjusted *p* values are presented. Post hoc analysis revealed more impairments in the i-autism and WS groups than in the ADHD group for all the ADI-R domain scores. There were no significant differences between the i-autism group and the WS group. The WS group alone showed more developmental delays than did the i-autism and i-ADHD groups.

**Table 2:** Group comparisons of autistic symptoms measured by the ADI-R and ADOS-2.

ADI-R domain total	WS (N = 24)	Autism (N = 24)	ADHD (N = 24)	Kruskal‒Wallis *Χ*^*2*^ (df = 2)^a^	${E}_{R}^{2}$	Post hoc comparisons^b^		
	Mdn (MAD) range	Mdn (MAD) range	Mdn (MAD) range			WS—autism	WS > ADHD	Autism > ADHD
ADI-R—during development								
A Social	8.0 (3.5) 1–22	9.0 (4.5) 0–28	2.0 (2.0) 0–7	29.86	0.421		+	+
B Communication	7.5 (2.5) 3–15	8.0 (3.0) 0–21	2.0 (2.0) 0–8	29.37	0.414		+	+
C RRB	3.0 (1.0) 0–8	3.0 (1.0) 0–9	0.0 (0.0) 0–5	27.48	0.387		+	+
D Developmental delay	3.0 (0.5) 2–5	1.0 (1.0) 0–5	1.0 (1.0) 0–5	24.21	0.341	+ (WS > autism)	+	
ADI-R—adolescence/adulthood								
A Social	4.0 (2.5) 0–12	5.0 (3.0) 0–14	0.0 (0.0) 0–9	25.90	0.365		+	+
B Communication	5.0 (2.0) 0–10	3.5 (2.5) 0–11	1.0 (1.0) 0–5	24.20	0.341		+	+
C RRB	2.5 (1.5) 0–11	2.0 (1.0) 0–10	0.0 (0.0) 0–5	21.08	0.297		+	+
ADOS-2—adolescence/adulthood								
Social affect	2.5 (0.5) 1–8	7.0 (3.0) 2–18	2.0 (1.0) 0–6	33.55	0.479	+ WS < autism		+
RRB	0.0 (0.0) 0–3	2.0 (1.0) 0–6	(0.0) 0–2	19.52	0.279	+ WS < autism		+
Severity	1.0 (1.0) 1–6	5.0 (2.0) 2–10	1.5 (0.5) 1–3	34.00	0.486	+ WS < autism		+

*ADI-R* Autism Diagnostic Interview-Revised, *ADOS-2 * Autism Diagnostic Observation Schedule, 2nd Edition, *Mdn*  median, *MAD* median absolute distance, *WS* Williams syndrome, *ADHD* attention deficit/hyperactivity disorder, *RRB* repetitive and restricted behavior.

^a^All *F* = *p* < .001, ^b^all games Howell post hoc analyses = *p* < 0.001, +  = significant difference.

On the item level, there were four items included in the diagnostic algorithm, in which a substantial part of the WS group showed impairments during development, i.e., > 40% of the sample scored > 0. These items were related to lack of shared enjoyment (i.e. “Showing and directing attention”, “Offering to share”, “Seeking to share enjoyment with others”, “Offering comfort”, “Quality of social overtures”), relative failure to initiate and sustain conversational interchange (i.e. “Social verbalization/chat”, and “reciprocal conversation”), challenges in social awareness (i.e., “Inappropriate questions”*)*, and RRB* (*i.e.*,* “Unusual preoccupations” and “Repetitive use of objects/interests in parts of objects”). The results for all items are provided in the supplementary material (Tables S2-S4).

#### ADOS-2

**Figure 1:**
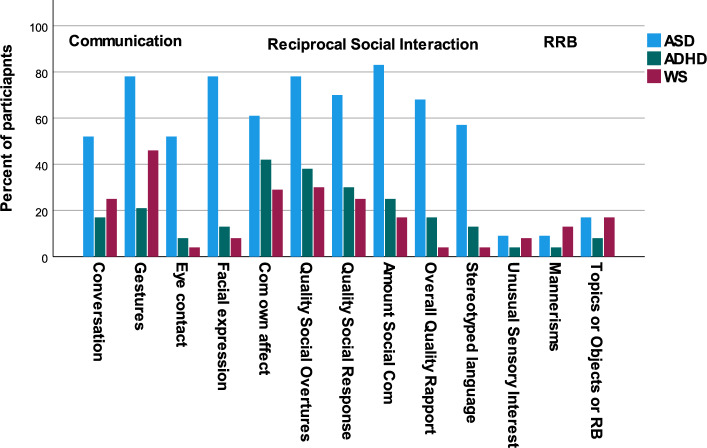
The figure includes the ADOS-2 algorithm items included in both modules 3 and 4, and the bars show the percentage of participants with a score of 1 or above, denoting presence of autism characteristics.

The Kruskal‒Wallis H test was used to determine if there were group differences in ADOS-2 scores (Table 2). Post hoc analysis (Dunn's procedure with a Bonferroni correction for multiple comparisons) revealed more symptoms and more severe impairments in the i-autism group, than in the WS and i-ADHD groups, on all the ADOS-2 domains and total scores. There were no significant differences between the WS and i-ADHD groups. Figure 1 shows the differences in item scores on the ADOS-2.

#### SRS-2

**Figure 2:**
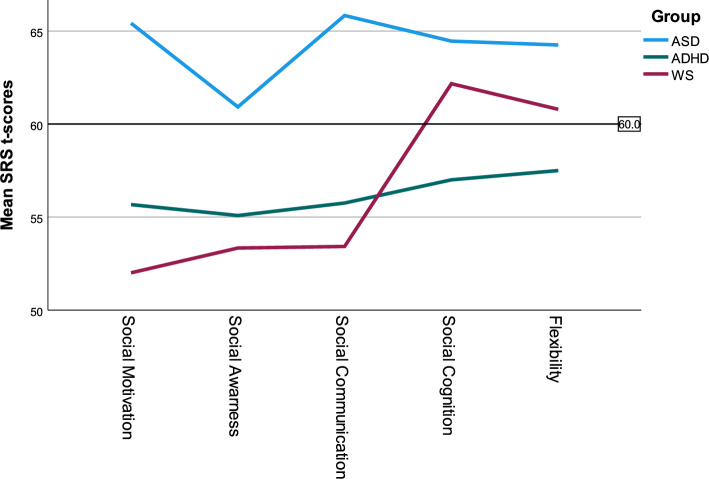
SRS-2 profiles in each diagnostic group.

The descriptive data of the raw SRS-2 raw scores are presented in Table 3. The SRS-2 total T scores in the WS group were below the cut-off for clinically significant difficulties (M = 57.2, SD = 13.8). The results from the subscales showed that the WS group was rated above the cut-off for mild-to-moderate difficulties on RRB (M = 60.8, SD = 14.5) and social cognition (M = 62.2, SD = 13.6), while communication (M = 53.4, SD = 13.6), social awareness (M = 53.3, SD = 13.3), and social motivation (M = 52.0, SD = 10.7) were rated clearly below the cut-off for any difficulties; see Fig. 2.

**Table 3:** Group comparisons of parent-reported autistic traits on the SRS-2.

SRS-2 raw scores	WS (N = 24)	Autism (N = 24)	ADHD (N = 24)	ANOVA *F *(df = 2)	*p*	ƞ^2^	Post hoc comparisons		
	M (SD) range	M (SD) range	M (SD) range				Autism > WS	WS > ADHD	Autism > ADHD
Total	64.29 (27.53) 21–124	83.33 (22.78) 21–132	47.67 (21.67) 9–99	13.131	< 0.001	0.276	+		+
RRB	13.08 (6.80) 3–27	14.17 (7.06) 3–32	7.67 (3.98) 2–16	7.801	< 0.001	0.184		+	+
Social cognition	14.09 (5.97) 5–26	15.00 (5.80) 0–27	8.92 (5.85) 0–23	8.543	< 0.001	0.198		+	+
Social communication	17.83 (9.97) 3–41	27.88 (8.32) 11–46	14.75 (8.27) 1–37	14.316	< 0.001	0.293	+		+
Social awareness	8.21 (3.91) 1–16	10.33 (4.14) 4–20	7.67 (3.58) 1–13	3.162	0.049	0.084			+
Social motivation	10.21 (5.23) 1–19	15.96 (5.50) 3–25	8.67 (4.35) 2–20	13.893	< 0.001	0.287	+		+

+   = significant difference.

*SRS-2*  social responsiveness scale, 2nd edition, *WS* Williams syndrome, *ADHD*  attention deficit/hyperactivity disorder.

One-way ANOVA was conducted to determine whether the SRS-2 scores differed among the three diagnostic groups. Tukey post hoc analysis revealed that the parents in the i-autism group reported higher total scores, i.e., more autistic traits, than did the parents in the WS and i-ADHD groups. However, the WS participants also had significantly greater scores on the RRB and social cognition subscales, than did the i-ADHD group. The i-autism group showed more difficulties than both the WS and the i-ADHD groups on the Social Communication and Social Motivation subscales. There was a borderline significant difference between the i-autism group and the i-ADHD group on the Social Awareness subscale (*p* = 0.049) (see Table 3).

### Developmental trajectories

Comparing retrospective and current reports in the ADI-R, we found that social interaction and communication abilities improved over time in all three groups, i.e., there were fewer behavioural difficulties in adolescence/adulthood than during development. The Wilcoxon signed rank test showed that the decrease in symptom severity was significant for the social and communication domains (*p* < 0.05) in all groups, while there was no significant change in RRB symptoms (*p* > 0.05). In the WS group, there was a 46% decrease in social symptoms and a 42% decrease in communication symptoms. Further, there was a 49% decrease in social symptoms and a 47% decrease in communication symptoms in the i-autism group. In the i-ADHD group, there was a 45% decrease in social symptoms and a 50% decrease in communication symptoms.

Next, the items included in the diagnostic ADI-R algorithm were analysed to examine whether specific items or symptom domains of symptoms were more dominant in the change over time in the three groups. One item about friendship asked about the age of 10–15 years and was therefore excluded from this analysis. In the WS group, two items were associated with a significant decrease in symptom severity over time: “Offering to share” (*Z* = −2.270, *p* = 0.038) and “Quality of social overtures” (*Z* = −2.121, *p* = 0.034) (i.e., difficulties in these areas decreased over time). In the i-autism group, the items “Offering to share” (*Z* = −2.226, *p* = 0.026), “Quality of social overtures” (Z = −2.333, *p* = 0.020), and “Reciprocal conversation” (Z = −2.121. *p* = 0.034), showed a decrease in symptom severity over time. Finally, in the i-ADHD group, there was only one item that showed a significant decrease over time—i.e., “Neologisms/idiosyncratic language” (*Z* = −2.333, *p* = 0.020). For more details on the item analyses, see the supplementary material (Tables S2-S4).

### Social cognition and social approach behaviour

The Kruskal‒Wallis H test was used to determine whether diagnostic group had an effect on the scores of the selected items of interest (see supplementary Table S5). The scores were significantly different between the three groups for all items except for the parent-rated items “Quality of social overtures” and “Social disinhibition”. Pairwise comparisons were performed using Dunn’s procedure with a Bonferroni correction for multiple comparisons, and adjusted p values are presented. The post hoc analyses revealed that the WS group had greater difficulty than both comparison groups did on one item, “Inappropriate questions or statements”. Furthermore, the WS group was rated to have difficulties at the same level as the i-autism group, and significantly more than the i-ADHD group, on the following items: “Recognize when others try to take advantage of him or her”, “Awareness of standing close to someone”, and “Inappropriate questions or statements”. The i-autism group exhibited significantly more impairments than did the other two groups in terms of “Eye contact”, “Quality of social overtures”, “Amount of social overtures”, and “Ability to understand the meaning of other people’s tone or voice and facial expression”.

## Discussion

In the present study, autistic traits and symptoms in individuals with WS were examined in a transdiagnostic and multi-rater design. The results revealed syndrome-specific profiles of social strengths and difficulties in adolescents and adults with WS. The relative strengths associated with WS were high social motivation and a high frequency of social overtures, as well as typical nonverbal communication, such as social smiling, eye contact, and range of facial expressions. Of the included WS individuals, 12.5% met the criteria for an autism diagnosis. Nevertheless, a majority of WS individuals showed significant atypicality regarding the quality of social overtures, social inhibition, social verbalization, reciprocal conversation, and a lack of gestures. In addition, RRBs, such as repetitive use of objects and unusual preoccupations, were common in the WS group. There was a developmental trajectory in the WS group, with a decrease in autistic symptoms over time, similar to the developmental trajectory found in the comparison groups. Finally, the ADI-R and SRS-2 were more sensitive than the ADOS-2 for detecting autistic symptoms in individuals with WS.

Our results based on the ADI-R indicate that the WS group displays similar overall levels of autistic traits and symptoms as the i-autism group, and significantly greater levels than the i-ADHD group, regarding the quality of communication, and the quality of social interaction and reciprocity. In particular, a lack of shared enjoyment was common in the WS group, such as an inadequacy of showing and directing attention, offering to share, and offering to comfort*.* Furthermore, a majority of WS individuals displayed atypical qualities of social overtures and difficulties with initiating and sustaining reciprocal conversations. These results are in line with previous research on conversational skills, which indicated that children with WS had more difficulties with pragmatic language skills than children with Down syndrome and a similar intellectual level (i.e., inappropriate initiation of conversation and the use of stereotyped conversation)^7^. Our results add to prior findings by suggesting that, in comparison to individuals with common neurodevelopmental conditions, individuals with WS are more prone to ask inappropriate questions and make inappropriate remarks in conversations. However, in comparison to those in the i-autism group, the WS group exhibited less stereotyped language, which was supported by the findings of both the ADI-R and the ADOS-2.

In contrast to those in the i-autism group, and at the same level as those in the i-ADHD group, the WS group demonstrated more social overtures. These findings are in line with previous findings of an increased social drive associated with the disorder^57^, and an intense desire to form affective bonds with others^58^. Furthermore, the WS group exhibited nonverbal communication behaviours, such as social smiling, eye contact, and range of facial expressions, which are typically altered in individuals with i-autism. These abilities might be linked to the elevated empathy consistently reported in WS^59^. However, the results also showed a lack of gestures associated with WS. This has previously been reported in toddlers and children with WS ^25,27^, and our results indicate that this challenge persists during adolescence and adulthood.

The phenotypes of WS and autism might be described as opposite extremes of social approach behaviour. Nevertheless, both groups have specific combinations of socio-cognitive difficulties and atypical cognitive and intellectual abilities, making them more likely to be easy targets for victimization. As a result, both groups have a highly elevated risk of being victims of predatory behaviours such as bullying and abuse, in real-life settings as well as online^60^. For example, sexual abuse is reported in 18–20% of individuals with i-autism and WS^61^. One of the most common concerns of parents of individuals with WS is that their child’s atypical approach behaviour and consistent interest in strangers will make them socially vulnerable^62^. Individuals with WS have more difficulty recognizing others’ negative intentions than do other groups with a similar intellectual level^35^. Neurobiological underpinnings have been proposed by studies showing less arousal and decreased amygdala activity in response to threatening social stimuli, such as angry faces, in comparison to control groups^15,63^. In the present study, parents reported that 92% of the WS individuals had difficulties recognizing when others tried to take advantage of them, in comparison to 67% with i-autism and 54% with i-ADHD. Due to the major effects that these difficulties have on an individual level, interventions targeting the specific combination of social cognition difficulties, intellectual profiles, and social approach behaviours in individuals with WS ought to be a future research priority.

Furthermore, in the present study, both the WS and i-autism groups showed similar difficulties regarding personal space awareness, and more than in the i-ADHD group. The same item on the SRS-2, i.e., “Lacking awareness of standing to close to someone”, was analysed in a transdiagnostic design in a large sample of individuals with WS and i-autism, suggesting that the individuals with WS had less awareness of the personal sphere than autistic individuals did^30^. A possible explanation for the discrepancy in the results between the study by Lough et al. and our study, is that the WS sample in Lough et al*.* study was younger (mean age of 13.5 years) than that in the present study. We speculate that there might be different developmental trajectories regarding social disinhibition in WS than in i-autism, being more pronounced in young children with WS and decreasing with age to a similar level as in i-autism. Another example of this is “inappropriate questions” in the ADI-R, in which 67% of individuals in the WS sample experienced challenges during development, 54% in adolescents and adults (a borderline significant change; *p* = 0.058), and 38% (during development) and 25% (in adolescence and adulthood) in the i-autism group. However, this needs to be further investigated in future studies.

In line with the findings of previous research^5,6,29,31,64^, our results from the SRS-2 suggest the presence of WS-specific profiles of social functioning. Individuals with WS exhibit prosocial behaviours in areas of social communication, social awareness and social motivation in contrast to individuals with i-autism, which typically present with clinically significant difficulties in all five SRS-2 domains. However, individuals with WS still encounter notable difficulties in social cognition and RRB.

The discrepancy between elevated social motivation and a desire for social contact, and the difficulties in social cognition and flexibility, are presumably key components of the social challenges experienced by individuals with WS. As mentioned above, the autistic features associated with WS might be captured by Wing and Gould’s description of the “active but odd” autism subtype^17^, i.e. actively seeking interaction with others, although in unusual ways. Research has also shown that the “active but odd” group is associated with milder autistic symptoms, greater verbal ability, and greater risk for ADHD, ODD, and other socioemotional problems, in comparison with other autistic individuals^65^. Future studies using behavioural measures, which are intended to particularly capture different subtypes of autism such as “active but odd”, might be informative for WS research.

Our findings suggest a developmental trajectory for social interaction and communication difficulties in which symptom severity decreases with increasing age in the WS and comparison groups. In autism, the literature indicates that IQ is the strongest predictor of outcome and symptom improvement over time, and that individuals with co-occurring autism and intellectual disability show less change over time than individuals diagnosed with autism and IQ within the normal range^66^. Although the WS individuals in our study had a significantly lower mean IQ than did those in the comparison groups, and 91.7% met the criteria for intellectual disability vs 16.7% in the i-autism group, our results indicate no group difference in the degree to which autistic symptoms develop over time. Thus, our results suggest that there might be different predictors of trajectories regarding social and communicative abilities in WS than in i-autism.

Our results show a discrepancy between the parent-reported ADI-R scores and the observed and expert rated ADOS-2 scores in the WS group. Parents of individuals with WS, reported more difficulties (i.e., at the same level as the i-autism group) than did the observation ratings provided by experts (i.e., significantly lower than the i-autism group). These results suggest that the ADI-R and the ADOS-2 capture symptoms of autism differently in individuals with WS than in those with i-autism. Research on different subgroups of autism, such as the previously mentioned active-but-odd subgroup, has shown a positive association with SRS scores but a negative association with ADOS-2 scores^65^. We speculate that this might be because the social challenges in individuals with WS are more subtle than those in individuals with i-autism. Since symptoms need to be observed during the limited time of administration in the ADOS-2, it is less sensitive than the ADI-R or the SRS-2 for identifying such subtle difficulties. Parents on the other hand have a more in-depth picture of their children’s behaviour across different contexts and over a longer period of time, than psychologists can obtain during observation sessions. Based on our results, we argue that whereas the combination of the ADOS-2 and ADI-R is the gold standard for the diagnostic assessment of i-autism, the ADI-R and SRS-2 are more sensitive at detecting social difficulties in individuals with WS. Nevertheless, neither the ADI-R nor the SRS-2 are designed to capture the social deficits specific to WS, and the development of less autism-specific instruments suitable for the assessment of social impairments in other diagnostic groups, such as WS, ought to be a future research and clinical priority.

The current study is not without limitations. Although a powerful transdiagnostic design is used^8,67^, the sample sizes of each diagnostic group are still relatively small, which limits the statistical power to detect small to moderate differences between the groups. Accordingly, we could not compare subgroups of WS individuals, such as WS individuals with and without autism. Another limitation related to the comparison groups is the group differences in intellectual ability. On the other hand, alternative approaches such as (i) including a comparison group of individuals with idiopathic intellectual disability, or (ii) a cross-syndrome design, comparing WS with other genetic syndromes with phenotypes of intellectual disability such as Fragile X syndrome or Down syndrome, also have limitations. First, an idiopathic intellectual disability group would have been highly heterogeneous, most likely including several different genetic syndromes not yet identified, making the results unspecific. Second, other genetic syndromes have their own specific profiles of autism symptoms, limiting the conclusions that can be drawn from cross-syndrome designs to cross-syndrome differences. Instead, in the present study, autism diagnoses were based on cognitive testing, adaptive profiles, other psychiatric comorbidities, and gold-standard measures of autism (for more details, see^36^) to facilitate differentiation between intellectual ability and autism symptomatology. In addition, we analysed the associations between IQ and specific items related to autism (see supplementary material), but still, we cannot rule out that IQ interferes with the autistic symptoms that we aimed to assess. As expected, there were group differences in co-occuring psychiatric conditions such as a higher prevalence of anxiety in the WS group, in comparison to the i-autism and i-ADHD groups^4^. More surprisingly, two of the individuals in the WS group met criteria for psychotic disorders (i.e. brief psychotic disorder and psychotic disorder not otherwise specified). Although, consensus diagnoses were made based on all available information, we can not rule out that these psychotic symptoms affected the behavioral measures collected from these individuals. These results further indicate that psychotic symptomatology, might be an important area for future WS research.

Moreover, there was a significant difference in age between the WS group and the comparison groups, with the WS group being older than the i-autism and i-ADHD groups. Since our results indicate a negative association between age and some of the outcome measures (i.e. SRS-2 and ADI-R RRB scores), as well as a decrease in symptom severity over time, this age difference might have affected the group comparisons. Possibly, a younger WS group would have shown more autism symptoms. This could also explain the relatively low prevalence of autism diagnoses in the WS group, in comparison to previous studies based on children (e.g.^27^).

Finally, 93% of the participants’ parents were of Scandinavian origin, in comparison to ~ 20% of the total Swedish population being born outside Scandinavia^69^. Hence, as in most clinical research there is a potential selection bias, considering that families with higher socioeconomic status and that are well integrated into society, are more likely to participate in research studies.

First-choice clinical scales for the assessment of autism were used; however, these measures still have some limitations. Since our study was cross-sectional, we used retrospective ratings of historical symptoms in the ADI-R, which might have induced recall bias. In particular, parent-provided historical data on symptom onset and regression has shown to likely be biased^70^. Furthermore, the historical scores of the ADI-R are a combination of early symptoms between 4 and 5 years of age and “ever”, limiting the possibility of detecting developmental trajectories. Although the SRS-2 is one of the most commonly used instruments for measuring autistic traits in the WS literature, the five-factor model of the instrument has been questioned. Hence, interpretations of the analyses based on the SRS-2 subscale scores need to be performed with caution.

Despite these limitations, our results add to the literature and shed further light on the social phenotype of WS. The results emphasize the need for clinical assessments of autistic symptoms in individuals with WS, as well as the need for valid measures and interventions targeting the specific social challenges associated with the disorder. Furthermore, the autism research field is limited by the following: (i) most studies exclude participants with intellectual disability, and (ii) most autism diagnostic and screening instruments, are less specific in individuals with intellectual disability, than in individuals within the normal range of intellectual ability^68^. Therefore, our study also contributes to the general understanding of the autism phenotype and its subtypes.

### Supplementary Information

Supplementary Tables.

## Data Availability

Anonymized data will be made available to researchers upon reasonable request from the corresponding author (charlotte.willfors@ki.se).
