# Supplementary material for: Symptoms of autism in Williams syndrome: a transdiagnostic approach
Source: Sci Rep. 2024 Jul 30;14:17583. doi: 10.1038/s41598-024-68089-0 (PMC11289373; doi:10.1038/s41598-024-68089-0)
Supplement: Supplementary file 1 — Supplementary Tables. [file 41598_2024_68089_MOESM1_ESM.pdf]

Supplementary material

Table S1. Correlations between total IQ and measures of autistic traits and symptoms.

	WS (N=24)		Autism (N=24)		ADHD (N=24)	
	r_s	*p*	r_s	*p*	r_s	*p*
**ADI-R current**						
A Social	-.073	.733	-.519	**.011**	-.346	.097
B Communication	-.328	.118	-.244	.262	-.383	.065
C RRB	-.302	.152	-.066	.763	-.113	.600
**ADOS-2**						
Social Affect	-.202	.345	-.007	.975	-.196	.359
RRB	-.455	**.026**	.533	**.011**	-.032	.353
Severity	-.343	1.00	.141	.533	-.092	.149
**SRS-2**						
Total	-.177	.409	-.337	.116	-.561	**.004**

Note: Bold typeface indicate $p<.05$.

Table S2. Autistic symptoms during development vs current on ADI-R items in the WS group

	WS sample (n=24)						Wilcoxon Z	*p*
**ADI-R items[a]**	**During development**			**Adolescence/Adulthood**				
**Coding[b]**	0	1	≥ 2	0	1	≥ 2		
**A1: Failure to use nonverbal behaviour to regulate social interaction**								
Social smiling	75%	21%	4%	75%	21%	4%	0	1.000
Range of facial expression	83%	13%	4%	80%	21%	0	0	1.000
**A2: Lack of shared enjoyment**								
Showing and directing attention	46%	4%	50%	42%	33%	25%	1.311	.190
Offering to share	54%	25%	21%	38%	25%	38%	**2.271**	**.023**
Seeking to share enjoyment with others	58%	21%	21%	58%	29%	13%	1.000	.317
**A3: Lack of socioemotional reciprocity**								
Use of other's body to communicate	100%	0%	0%	100%	0%	0%	0	1.000
Offering comfort	58%	33%	8%	67%	33%	0%	1.414	.157
Quality of social overtures	58%	38%	4%	80%	21%	0%	2.121	.034
Inappropriate facial expression	83%	8%	8%	88%	8%	4%	1.000	.317
Appropriateness of social responses	75%	13%	13%	79%	13%	8%	1.000	.317
**B1: Lack of, or delay in, spoken language and failure to compensate through gestures**								
Pointing to express interest	71%	21%	8%	67%	21%	13%	.816	.414
Nodding	67%	17%	17%	58%	21%	21%	1.000	.317
Head shaking	79%	4%	17%	62%	17%	21%	1.890	.059
Conventional/instrumental gestures	63%	25%	13%	63%	21%	17%	.447	.655
**B2: Relative failure to initiate or sustain conversational interchange**								
Social verbalization/chat	54%	21%	25%	54%	21%	25%	0	1.000
Reciprocal conversation	46%	25%	29%	46%	25%	29%	0	1.000
**Stereotyped, repetitive, or idiosyncratic speech**								
Stereotyped utterances	78%	13%	9%	83%	9%	9%	1.000	.317
Inappropriate questions	33%	46%	21%	46%	46%	8%	1.897	.058
Pronominal reversal	88%	13%	0%	92%	4%	4%	.000	1.000
Neologisms/idiosyncratic language	96%	4%	0%	96%	4%	0%	.000	1.000
**C1: Encompassing preoccuation or circumscribed pattern of interest**								
Unusal preoccupations	54%	38%	8%	58%	38%	4%	1.000	.317
Circumscribed interests	79%	13%	8%	79%	13%	8%	0	1.000
**C2: Apparently compulsive adherence to non-functional routines or rituals**								
Verbal rituals	96%	0%	4%	96%	0%	4%	0	1.000
Compulsions/rituals	63%	25%	13%	63%	21%	17%	1.000	.317
**C3: Stereotyped and repetitive motor mannerisms**								
Hand and finger mannerisms	63%	25%	13%	75%	21%	4%	-1.890	.059
Other complex mannerisms/ stereotyped body movements	67%	21%	13%	79%	13%	8%	-1.633	.102
**C4: Preoccupations with part of objects or non-functional elements of materials**								
Repetitive use of objects/interests in parts of objects	50%	29%	21%	67%	17%	17%	1.667	.096
Unusual sensory interests	63%	29%	8%	71%	29%	0%	1.633	.102

a. The items are based on the current algorithm for individuals age >10 years since only one participant were of age under 10 years.
b. 0=Behaviour was not present, 1=Behaviour was present in an abnormal form (or "lack of behaviour"), but not sufficiently severe, frequent, or marked to meet the criteria for "2", 2/3=Definite abnormality of the type specified

Note: Bold typeface indicate p<.05.

Table S3. Autistic symptoms during development vs current on ADI-R items in the i-autism group

	i-autism (n=24)						Wilcoxon Z	*p*
**ADI-R items**	**During development**			**Adolescence/Adulthood**				
**Coding[a]**	0	1	≥ 2	0	1	≥ 2		
**A1: Failure to use nonverbal behaviour to regulate social interaction**								
Social smiling	58%	21%	21%	44%	39%	17%	-.905	.366
Range of facial expression	58%	13%	29%	58%	29%	13%	-1.100	.271
**A2: Lack of shared enjoyment**								
Showing and directing attention	63%	17%	21%	79%	13%	8%	-1.413	.158
Offering to share	50%	17%	33%	71%	17%	13%	**-2.226**	**.026**
Seeking to share enjoyment with others	54%	25%	21%	54%	38%	8%	-1.000	.317
**A3: Lack of socioemotional reciprocity**								
Use of other’s body to communicate	88%	13%	0%	96%	4%%	0%	-1.633	.102
Offering comfort	42%	25%	33%	58%	21%	21%	-1.811	.070
Quality of social overtures	50%	33%	17%	65%	30%	4%	**-2.333**	**.020**
Inappropriate facial expression	71%	25%	4%	75%	21%	4%	-.272	.785
Appropriateness of social responses	42%	25%	33%	54%	33%	13%	-1.710	.087
**B1: Lack of, or delay in, spoken language and failure to compensate through gestures**								
Pointing to express interest	63%	25%	13%	67%	21%	13%	-.302	.763
Nodding	75%	17%	8%	83%	17%	0%	-1.300	.194
Head shaking	71%	21%	8%	83%	13%	4%	-1.300	.194
Conventional/instrumental gestures	63%	8%	29%	58%	25%	17%	-.632	.527
**B2: Relative failure to initiate or sustain conversational interchange**								
Social verbalization/chat	17%	38%	46%	29%	33%	38%	-1.890	.059
Reciprocal conversation	25%	38%	38%	38%	38%	25%	**-2.121**	**.034**
Stereotyped, repetitive, or idiosyncratic speech								
Stereotyped utterances	58%	25%	17%	67%	17%	17%	-1.414	.157
Inappropriate questions	63%	25%	13%	75%	25%	0%	-1.730	.084
Pronominal reversal	79%	8%	13%	92%	4%	4%	-1.633	.102
Neologisms/idiosyncratic language	79%	13%	8%	96%	4%	0%	-1.857	.063
**C1: Encompassing preoccuation or circumscribed pattern of interest**								
Unusal preoccupations	88%	13%	0%	21%	8%	0%	-1.000	.317
Circumscribed interests	38%	25%	38%	39%	30%	30%	-1.732	.083
**C2: Apparently compulsive adherence to non-functional routines or rituals**								
Verbal rituals	83%	13%	4%	88%	8%	4%	-1.000	.317
Compulsions/rituals	50%	13%	38%	58%	13%	29%	-1.633	.102
**C3: Stereotyped and repetitive motor mannerisms**								
Hand and finger mannerisms	83%	17%	0%	88%	4%	8%	-1.089	.276
Other complex mannerisms/ stereotyped body movements	79%	21%	0%	89%	17%	0%	-1.000	.317
**C4: Preoccupations with part of objects or non-functional elements of materials**								
Repetitive use of objects/interests in parts of objects	75%	17%	8%	79%	21%	0%	-1.732b	.083
Unusual sensory interests	83%	13%	4%	83%	13%	4%	0	1.000

a. 0=Behaviour was not present, 1=Behaviour was present in an abnormal form (or “lack of behaviour”), but not sufficiently severe, frequent, or marked to meet the criteria for “2”, 2/3=Definite abnormality of the type specified.
Note: Bold typeface indicate p<.05.

Table S4. Autistic symptoms during development vs current on ADI-R items in the i-ADHD group

	i-ADHD (n=24)						Wilcoxon Z	*p*
**ADI-R items**	**During development**			**Adolescence/Adulthood**				
**Coding[a]**	0	%1	≥ 2	0	1	≥ 2		
**A1: Failure to use nonverbal behaviour to regulate social interaction**								
Social smiling	88%	13%	0%	83%	13%	4%	-1.414	.157
Range of facial expression	100%	0%	0%	92%	8%	0%	-1.414	.157
**A2: Lack of shared enjoyment**								
Showing and directing attention	100%	0%	0%	92%	8%	0%	-1.414	.157
Offering to share	67%	13%	21%	83%	4%	13%	-1.656	.098
Seeking to share enjoyment with others	100%	0%	0%	100%	0%	0%	0	1.000
**A3: Lack of socioemotional reciprocity**								
Use of other's body to communicate	96%	4%	0%	88%	13%	0%	-1.000	.317
Offering comfort	88%	13%	0%	88%	13%	0%	0	1.000
Quality of social overtures	92%	8%	0%	92%	8%	0%	0	1.000
Inappropriate facial expression	88%	13%	0%	83%	17%	0%	-.577	.564
Appropriateness of social responses	88%	13%	0%	92%	8%	0%	-.577	.564
**B1: Lack of, or delay in, spoken language and failure to compensate through gestures**								
Pointing to express interest	88%	8%	4%	96%	4%	0%	-1.342	.180
Nodding	96%	4%	0%	92%	4%	4%	-1.000	.317
Head shaking	96%	4%	0%	88%	8%	4%	-1.414	.157
Conventional/instrumental gestures	96%	4%	0%	88%	13%	0%	-.577	.564
**B2: Relative failure to initiate or sustain conversational interchange**								
Social verbalization/chat	79%	13%	8%	75%	21%	4%	0	1.000
Reciprocal conversation	88%	8%	4%	88%	8%	4%	0	1.000
Stereotyped, repetitive, or idiosyncratic speech								
Stereotyped utterances	88%	13%	0%	88%	13%	0%	.000	1.000
Inappropriate questions	79%	17%	4%	100%	0%	0%	-1.732	.083
Pronominal reversal	88%	8%	4%	100%	0%	0%	-1.633	.102
Neologisms/idiosyncratic language	75%	21%	4%	100%	0%	0%	-2.333	.020
**C1: Encompassing preoccuation or circumscribed pattern of interest**								
Unusal preoccupations	92%	8%	0%	96%	4%	0%	-1.000	.317
Circumscribed interests	79%	17%	4%	88%	8%	4%	-1.414	.157
**C2: Apparently compulsive adherence to non-functional routines or rituals**								
Verbal rituals	100%	0%	0%	100%	0%	0%	0	1.000
Compulsions/rituals	88%	4%	8%	92%	8%	0%	-1.000	.317
**C3: Stereotyped and repetitive motor mannerisms**								
Hand and finger mannerisms	100%	0%	0%	100%	0%	0%	0	1.000
Other complex mannerisms/ stereotyped body movements	100%	0%	0%	100%	0%	0%	0	1.000
**C4: Preoccupations with part of objects or non-functional elements of materials**								
Repetitive use of objects/interests in parts of objects	92%	8%	0%	100%	0%	0%	-1.414	.157
Unusual sensory interests	88%	13%	0%	83%	17%	0%	-.577	.564

a. 0=Behaviour was not present, 1=Behaviour was present in an abnormal form (or "lack of behaviour"), but not sufficiently severe, frequent, or marked to meet the criteria for "2", 2/3=Definite abnormality of the type specified.

Table S5. Observed and parent-reported autistic traits and symptoms on item level

Item	WS (N=24)			Autism (N=24)			ADHD (N=24)			Mean total IQ (n=72)			Kruskal-Wallis χ^2 (df=2)	*p*	Post hoc pairwise comparisons[e]		
**Answering alternatives**	0	1	≥ 2	0	1	≥ 2	0	1	≥ 2	0	1	≥ 2			**Autism - WS**	**WS> ADHD**	**Autism> ADHD**
**Observed impairments (ADOS-2)**																	
Eye contact.[a,b]	95%	-	5%	45%	-	55%	90%	-	11%	82	-	88	16.233	<.001	+ Autism>WS		+
Quality social overtures[a]	71%	29%	0%	26%	70%	4%	65%	35%	0%	78	88	88	11.554	.003	+ Autism>WS		+
Amount social overtures[a]	83%	17%	0%	35%	44%	17%	88%	13%	0%	80	88	90	20.334	<.001	+ Autism>WS		+
**Parent reported impairments (SRS-2, ADI-R Current)**																	
Eye contact, 4-5 years.	71%	21%	8%	42%	33%	25%	92%	4%	4%	83	81	79	13.575	.001	+ Autism>WS		+
Does not recognise when others try to take advantage of him or her.	8%	25%	67%	33%	25%	42%	46%	29%	25%	97	81	73	13.915	<.001		+	
Understand the meaning of other people's tone or voice and facial expressions.[d]	29%	38%	33%	8%	25%	67%	38%	42%	21%	76	83	82	12.821	.002	+ Autism>WS		+
Awareness of standing to close to someone[a]	13%	25%	63%	17%	38%	46%	50%	21%	29%	91	83	77	8.465	.015		+	
Inappropriate questions or statements.[a]	46%	46%	8%	75%	25%	0%	88%	13%	0%	87	76	48	10.871	.004	+ Autism<WS	+	
Quality of social overtures.[a]	79%	21%	0%	65%	30%	4%	92%	8%	0%	85	70	63	5.054	.080	-	-	-
Social disinhibition.[a]	46%	36%	18%	46%	29%	25%	63%	33%	4%	86	81	79	2.960	.228	-	-	-

a. ADOS-2/ADI-R: Higher values implies more impairments (e.g.,0=Behaviour was not present, 3=Definite atypicality of the type specified).

b. Only 0 and 2 are available as scoring options on this item. [c] SRS-2: Higher values implies more impairments (e.g. 0=Not true, 3=Almost always true), [d] For descriptive purpose, the item has been reversed. + = significant difference. Abbreviations: ADI-R=Autism Diagnostic Interview-Revised, ADOS-2=autism diagnostic observation scale, 2nd Edition, SRS-2=Social Responsiveness Scale, 2nd edition, WS=Williams syndrome, ADHD=Attention Deficit/Hyperactivity-Disorder.
